# DNA damage and repair in patients undergoing myocardial perfusion single-photon emission computed tomography

**DOI:** 10.1038/s41598-024-63537-3

**Published:** 2024-06-07

**Authors:** Andrea De Lorenzo, Maria Clara dos Santos Fernandes, Francisco Romeiro, Anna Paula Arpini, Glauber Monteiro Dias

**Affiliations:** 1 Coordenação de Ensino e Pesquisa, Instituto Nacional de Cardiologia, Rua das Laranjeiras 374, Rio de Janeiro, RJ Brazil; 2 Laboratório de Biologia Celular e Tecidual, Universidade Estadual do Norte Fluminense Darcy Ribeiro, Rio de Janeiro, Brazil; 3 Serviço de Medicina Nuclear, Instituto Nacional de Cardiologia, Rio de Janeiro, Brazil

**Keywords:** Myocardial perfusion imaging, Radiation, Comet assay, DNA, Cardiology, Health care, Medical research, Molecular medicine

## Abstract

As patient exposure to ionizing radiation from medical imaging and its risks are continuing issues, this study aimed to evaluate DNA damage and repair markers after myocardial perfusion single-photon emission computed tomography (MPS). Thirty-two patients undergoing Tc-99m sestamibi MPS were studied. Peripheral blood was collected before radiotracer injection at rest and 60–90 min after injection. The comet assay (single-cell gel electrophoresis) was performed with peripheral blood cells to detect DNA strand breaks. Three descriptors were evaluated: the percentage of DNA in the comet tail, tail length, and tail moment (the product of DNA tail percentage and tail length). Quantitative PCR (qPCR) was performed to evaluate the expression of five genes related to signaling pathways in response to DNA damage and repair (ATM, ATR, BRCA1, CDKN1A, and XPC). Mann–Whitney’s test was employed for statistical analysis; p < 0.05 was considered significant. Mean Tc-99m sestamibi dose was 15.1 mCi. After radiotracer injection, comparing post-exposure to pre-exposure samples of each of the 32 patients, no statistically significant differences of the DNA percentage in the tail, tail length or tail moment were found. qPCR revealed increased expression of BRCA1 and XPC, without any significant difference regarding the other genes. No significant increase in DNA strand breaks was detected after a single radiotracer injection for MPS. There was activation of only two repair genes, which may indicate that, in the current patient sample, the effects of ionizing radiation on the DNA were not large enough to trigger intense repair responses, suggesting the absence of significant DNA damage.

## Introduction

Due to its central role in the diagnostic and prognostic evaluation of coronary artery disease, myocardial perfusion imaging with single photon emission-computed tomography (MPS) is one of the most frequently performed noninvasive cardiac imaging tests, generating concern about the potentially excessive radiation exposure for patients, including the cumulative doses of lifetime radiation, even though the individual levels of radiation exposure are considered low^1,2^.

The understanding of the biological effects of low-dose radiation exposure is challenging. Most knowledge is derived from high-dose exposure, with the linear no-threshold (LNT) model definition that radiation risk is directly proportional to dose, and therefore any amount of radiation, even small, is potentially harmful. However, the validity of the LNT in the context of low radiation doses has been questioned^2,3^. Our group has verified that, after radiotracer injection for MPS, increased DNA fragmentation occurred, even though severe levels of damage were the least frequent^4^. With changes in imaging protocols that have led to a reduction of injected radiotracer doses, a re-evaluation of the genotoxic effects of ionizing radiation is desirable. Furthermore, it is known that in response to DNA damage, cells activate proteins and genes involved in apoptosis, DNA repair, cell cycle regulation, and chromatin remodeling, a process collectively known as the “DNA damage response” pathways^5^. The extent to which these pathways are activated may represent how patients respond to radiation exposure. This study sought to evaluate whether low dose radiation exposure from MPS induced DNA damage and the activation of repair pathways.

## Methods

Patients ≥ 18 years undergoing MPS were considered eligible for the study. Exclusion criteria were current or prior malignant neoplasm, autoimmune diseases, acute systemic diseases, significant trauma or major surgery in the past 3 months. The study was performed in line with the principles of the Declaration of Helsinki. Approval was granted by the Ethics Committee of the Instituto Nacional de Cardiologia (#67169717.6.0000.5272). Written, informed consent was obtained from all individual participants included in the study.

### Study protocol

Peripheral, venous blood samples (4 ml) were collected before and after radiotracer injection. Ethylenediaminetetraacetic acid (EDTA)-coated blood sampling tubes were used. Blood was collected in the morning in a nonfasting state, from 8–9 am (pre-exposure samples) and 11–12 am (post-exposure samples) and transported to the laboratory in a refrigerated container. MPS was performed with Tc-99m sestamibi (8 MBq/kg) in a 2-day protocol, but patients were evaluated only in the rest phase to avoid possible stress-induced effects on the DNA. The first peripheral blood sample (4 ml) was collected immediately before tracer injection, and the second, immediately before the patient left the Nuclear Cardiology laboratory after image acquisition (60–90 min after tracer administration). Images were acquired in a 2-head gamma camera (Infinia Hawkeye 4, General Electric Healthcare, WI, USA).

### Comet assay

Agarose-covered slides (1″ × 3″) were prepared in duplicates (two with blood collected before, and two with blood collected after tracer injection). Each slide received a mixture of 5 μl of whole blood and 120 μl of 0.5% low melting agarose (37 °C), was covered with a coverslide, and then refrigerated for five minutes. After agarose solidification, the coverslides were removed and the slides were incubated in a lysis solution (1% Triton X-100, 10% dimethyl sulfoxide, 2.5 M NaCl, 100 mM EDTA, 10 mM Tris) for two hours at 4 °C and protected from light. Peripheral blood cells, (PBCs), which became nucleoid structures after lysis, were analyzed.

The alkaline unwinding, electrophoresis and neutralization steps were performed as described by Hartmann and Speit^6^, with minor modifications. The slides were removed from the lysis solution and placed in the electrophoresis chamber, which was then filled with freshly made alkaline buffer (300 mM NaOH and 1 mM EDTA, pH 12.6). The cells were exposed to alkali for 40 min in an ice-bath to allow for DNA unwinding and the expression of alkali-labile sites. Subsequently, the DNA was submitted to electrophoresis for 30 min at 300 mA and 25 V (0.86 V/cm). All the above steps (preparation of slides, lysis and electrophoresis) were conducted without direct light in order to prevent additional DNA damage. Positive controls were performed with 200 ml of whole human blood, incubated for 2 h at 37 °C with 50 ml of methyl methanesulfonate (final concentrations of 0.08 mM and 0.016 mM). The two concentrations were used to demonstrate different levels of damage and to ascertain the assay sensitivity.

After electrophoresis, the slides were placed in a horizontal position and washed three times (5 min each) with 0.4 M Tris buffer, pH 7.5, to neutralize the excess alkali. Finally, slides were fixed with absolute ethanol, stained with GelRed 1:500 (Biotium) and analyzed using a fluorescence microscope at 40 × of magnification (Zeiss Axioplan with AxioCam MRc5 camera).

Images of fluorescence microscopy were analyzed using the Comet Assay Software Project Lab (CASPLab 1.2.3 beta 2). Three primary descriptors were evaluated to assess DNA damage: (1) the percentage of DNA in the tail, (2) tail length, and (3) tail moment, the product of the percentage of DNA in the tail and tail length. Two hundred nucleoids per sample were scored (100 nucleoids per slide).

### Gene expression analysis by quantitative PCR (qPCR)

Total RNA from the peripheral blood samples was extracted using the Maxwell^®^ RSC simplyRNA Blood Kit (Promega, WI, USA) in an automated Maxwell extractor (Promega, WI, USA). After normalization of RNA concentration from pre- and post-expositions samples, complementary DNA was synthesized with the Superscript IV Vilo Master Mix with EzDNAse Kit (Thermo Fisher Scientific, MA, USA).

The reverse transcription reaction was performed according to manufacturer´s instructions: 25 °C for 10 min, 50 °C for 10 min and 85 °C for 5 min. The reaction consisted of 4 µl of SuperScript™ IV VILO™ Master Mix, between 1 pg and 2.5 µg of RNA template, and up to 20 µl of nuclease-free water. The reaction was preceded by RNA quantification using spectrophotometry (260 nm) on NanoDrop ONE (Thermo Fisher Scientific).

Five genes related to signaling pathways in response to DNA damage and repair (ATM, ATR, BRCA1, CDKN1A, and XPC) were evaluated in this assay. Pre-tested primers for qPCR were obtained from Integrated DNA Technologies (IA, USA) and used at 500 nM in each reaction (Table 1). qPCR reactions were performed in triplicate in the real-time thermocycler StepOnePlus (Thermo Fisher Scientific, MA, USA) using the SYBR Green Power Track Master Mix (Thermo Fisher Scientific, MA, USA) reagent with the following cycles conditions: 95 °C for 20 s, 40 cycles of 95 °C for 3 s and 60 °C for 30 s. The comparison of 2^−ΔCt^ values of pre and post-exposure samples was used for the calculation of relative expression (RE). The reference gene was GAPDH.

**Table 1:** Primer sequences used in qPCR experiments.

GENE	Forward primer sequence 5′–3′	Reverse primer sequence 5′–3′
ATM	GAGCAGTCAGCAGAACTTGT	ACTGTCTCAGGAGTAGGAAGG
ATR	GAAGATGATGACCACACTGAGA	CCCAGACAAGCATGATCCAG
CDKN1A	GCAGACCAGCATGACAGAT	GAGACTAAGGCAGAAGATGTAGAG
BRCA1	AATGGAAGGAGAGTGCTTGG	ATACCTGCCTCAGAATTTCCTC
XPC	CAGCCAGTGAACAAGATAACCT	GAGCCCGGAGAATCAGTAAG
GAPDH	ACATCGCTCAGACACCATG	TGTAGTTGAGGTCAATGAAGGG

### Statistical analysis

The comet measuring and RE data were initially subjected to the Kolmogorov–Smirnov and Shapiro–Wilk tests to evaluate the normality of their distributions. As the condition was not met, the non-parametric Wilcoxon signed-rank test was employed to both experiments. Statistical analyses were conducted using GraphPad Prism 8. In all tests, the significance level was 5%.

## Results

Thirty-two patients were evaluated. The demographic and clinical characteristics of the study population, including the most frequent medications in use, are depicted in Table 2. The mean radiotracer dose was 15.1 mCi.

**Table 2:** Baseline characteristics of the study population.

Characteristic	
Age (years)	64.6 ± 9.0
Male	15 (47.0)
Diabetes	16 (50.0)
Dyslipidemia	19 (59.4)
Hypertension	27 (84.4)
Current smoking	2 (6.3)
Former smoking	8 (25.0)
Family history of CAD	17 (53.1)
Prior myocardial infarction	7 (21.9)
Prior PCI	6 (18.8)
Prior CABG	3 (9.4)
Cerebrovascular disease	1 (3.1)
Peripheral vessel disease	3 (9.4)
Medications	
Antiplatelet agents	21 (66.7)
Beta-blockers	27 (83.3)
ACEi/ARB	5 (15.6)

Numbers are n (%) or mean ± SD.

*ACEi/ARB* angiotensin-converting enzyme inhibitors/angiotensin receptor blockers, *CABG* coronary artery bypass grafting, *PCI* percutaneous transluminal coronary angioplasty.

### Comet assay

**Figure 1:**
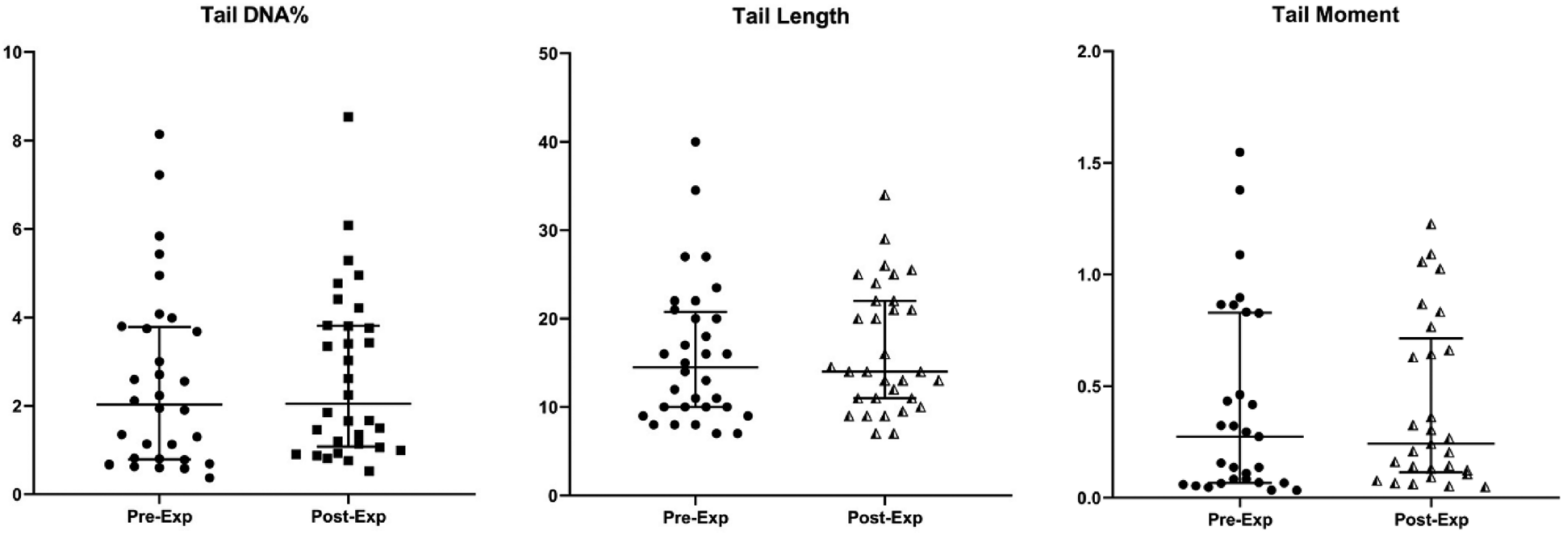
Distributions of medians of comet assay descriptors: percentage of DNA in the tail, tail length and tail moment (per subject before [Pre-Exp] and after [Post-Exp] exposure to ionizing radiation).

The medians of the descriptors of the nucleoids scored per sample (excluding outliers) were obtained for each patient. The comet descriptors were submitted to a per-patient analysis. The comparison between pre- and post-exposure medians of the 32 patients showed no significant difference for all descriptors (Fig. 1).

### Real-time qPCR

**Figure 2:**
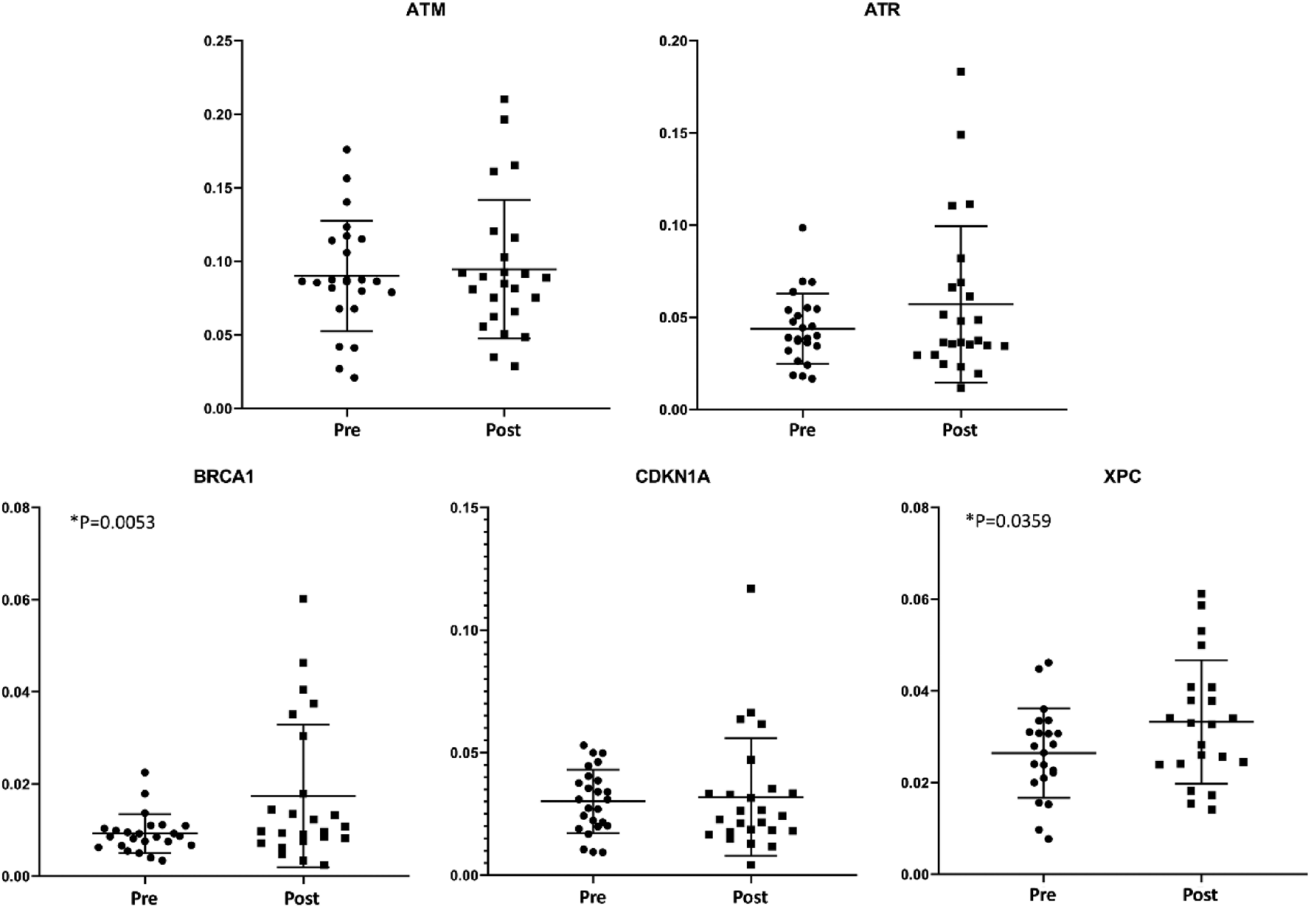
Expression of DNA damage response genes using qPCR. Box plot of delta Ct values in pre-exposure and post-exposure samples, per subject. *ATM* Ataxia Telangiectasia Mutated. serine/threonine protein kinase, *ATR* Ataxia Telangiectasia and Rad3-related protein, *BRCA1* Breast Cancer 1, *CDKN1A* Cyclin Dependent Kinase Inhibitor 1A, *XPC* Xeroderma Pigmentosum, Complementation Group C.

Gene expression analysis of the DNA damage and repair markers revealed an increase of BRCA1 and XPC expression after radiotracer injection (p = 0.0053 and 0.0359, respectively). The post-exposure expression of ATM, ATR and CDKN1A genes was not significantly different from pre-exposure samples (Fig. 2).

## Discussion

Exposure to ionizing radiation may be considered inherent to our lives; approximately 80% of our radiation exposure comes from background, naturally occurring sources, including radioactive materials found in the soil, water, and air, besides cosmic rays, especially at high altitudes^7^. The use of radiation for medical purposes represents 20% of the total population exposure^7^; nonetheless, the medical applications of ionizing radiation have been a matter of concern, even though, to date, there is no conclusive evidence that radiation from diagnostic imaging causes harm at the levels patients receive^3^.

In this study of patients undergoing MPS, using an automated analysis of the comet assay, in which peripheral blood cells were evaluated, we found no statistically different difference of DNA damage descriptors after radiotracer injection, comparing post-exposure to pre-exposure samples of each of the 32 patients.

Other authors have found evidence of DNA damage after radiotracer injection for nuclear medicine studies^8–10^. In a previous study by our group^4^, using visual analysis of DNA migration, the results showed an 18.3% increase in DNA damage after radiation exposure, although most cells (about 70%) remained free of DNA fragmentation after the radiotracer injection. However, in our prior study, the total amount of nucleoids was assessed, and a per-patient analysis was not performed. One may hypothesize that the larger numbers of nucleoids might have enabled the appearance of small differences, which are not evident in the current study, which focused on a per-patient, pre-post radiation exposure evaluation. Additionally, imaging protocols have been updated, resulting in lower radiotracer doses in current practice, which could generate different results regarding DNA damage.

Regarding DNA repair, it is known that when DNA damage occurs, there are self-protective mechanisms that promote repair, and cells activate repair genes^11^. In this study, using qPCR, only BRCA1 and XPC had increased expression after radiotracer injection, which could be interpreted as a possible low-level of activation of DNA repair genes secondary to DNA damage. Lee et al.^12^ also collected blood samples from patients (n = 63) before and after MPS and found no significant differences in the phosphorylation of biomarkers of DNA damage (H2AX, protein 53, and ataxia telangiectasia mutated), as well as either downregulated or unchanged expression in DNA damage response genes. López-Riego et al.^13^ analyzed peripheral blood mononuclear cells´ response to ionizing radiation. Gene expression of a panel of six radiation-responsive genes was measured qPCR before and 2 h after PET-CT (17 patients who had imaging with with ^18^F-Fluorodeoxyglucose, ^18^F-labelled prostate-specific membrane antigen or ^68^Ga-DOTA-Phe1 Tyr3-octreotate) or bone scintigraphy (17 patients injected with ^99m^Tc-methylene diphosphonate). None of the genes (BBC3*, *CDKN1A*, *FDXR*, *GADD45A*, *MDM2*, *XPC) was significantly upregulated at 2 h after tracer injection, compared to baseline. There were weak trends of upregulation of BBC3*, *XPC and GADD45A genes in both groups and of CDKN1A*, *FDXR and MDM2 in PET-CT patients only, showing a low level of induced DNA repair response. The authors also assessed the phosphorylation of γH2AX, which signals the presence of DNA double-strand breaks, a deleterious DNA lesion^14,15^. γH2AX had a weak response, indicating that radiation exposure from these diagnostic procedures induced a slight increase in DNA damage, consistent with the subtle DNA repair response.

Yan et al.^16^ have also demonstrated that exposure to ionizing radiation induced a significant increase in BRCA1 expression, involving post-transcriptional mechanisms, among others. BRCA1 is a tumor suppressor gene, often mutated in familial breast and ovarian cancer^17^, which is involved in cellular pathways that maintain genomic stability, including DNA damage-induced cell cycle checkpoint activation, DNA damage repair, protein ubiquitination, chromatin remodeling, as well as transcriptional regulation and apoptosis^18,19^. Cell cycle checkpoints monitor the chromatin status during the cell cycle, arresting the cycle if damage is detected, allowing cells to repair damaged DNA before resuming cell cycle progression^20^. BRCA1 acts at all stages of the DNA damage response, from replication error monitoring to prophylactic elimination of damaged cells. This could justify the early activation of its gene expression, even in exposure to low-dose ionizing radiation.

XPC is a candidate biomarker of ionizing radiation exposure^21–23^, which acts in the recognition of DNA damage before the initiation of Global Genome Nucleotide Excision Repair (GG-NER). This protein recognizes helix distortions throughout the genome caused by DNA lesions^24^. Therefore, this gene is expected to be activated early in DNA damage response, such as BRCA1 gene. Individuals with mutations in the XPC gene exhibit a strong susceptibility to skin cancer due to the failure to remove UV radiation-induced photoproducts^25^.

It is worth noting that diverse responses observed in different studies could be related to the assessment of different tests, which employ different radiotracers and therefore may promote different responses; to individual patients’ radiosensitivity issues^26^; or to the variable activation of the DNA damage responses following low doses^11^.

It should be emphasized that medical imaging can provide valuable information, and the use of low doses of ionizing radiation for imaging has not shown definite clinical evidence of harm. Nonetheless, it is essential to use the tests judiciously, as proper usage is crucial to minimize potential health hazards^27^. Adherence to safety guidelines^28^ helps keep a balance between the benefits and risks associated with ionizing radiation exposure. The ALARA (“As Low As Reasonably Achievable”) principle, a guide for radiation safety, is always worth to be reminded, in parallel with the AHARA concept of “As High As Reasonably Achievable” benefit of medical imaging^29^, as they foster the continuous optimization of protocols, tracers and equipment, leading to the reduction of radiation exposure.

### Limitations

DNA integrity might also be affected by medications, smoking, exposure to other sources of radiation, among other conditions; however, in a real-world study, those cannot be excluded. The absence of a significant activation of other genes besides BRCA1 might be related to the timing of blood sampling, which might be relatively short in relation to the exposure to ionizing radiation, not allowing enough time for the detection of changes in gene expression. Finally, defining whether DNA damage levels return to baseline or not was not possible due to the fact that blood sampling was performed only in the day of the test, without further (24–48 h) sampling.

## Conclusions

This study suggests that a single radiotracer injection for MPS is not associated with significant DNA damage. The activation of only two repair genes may indicate that any possible amount of DNA strand breaks is not large enough to trigger intense repair responses and might not result in significantly deleterious DNA damage.

## Data Availability

The datasets used and/or analysed during the current study available from the corresponding author on reasonable request.

## Abbreviations

ACEi: Angiotensin-converting enzyme inhibitors
ARB: Angiotensin receptor blockers
ATM: Ataxia telangiectasia mutated. Serine/threonine protein kinase
ATR: Ataxia telangiectasia and Rad3-related protein
BRCA1: Breast cancer 1
CABG: Coronary artery bypass grafting
CDKN1A: Cyclin dependent kinase inhibitor 1A
DMSA: Dimercaptosuccinic acid
EDTA: Ethylenediaminetetraacetic acid
GAPDH: Glyceraldehyde 3-phosphate dehydrogenase
MPS: Myocardial perfusion single-photon emission computed tomography
NCRP: National council on radiation protection and measurements
PCI: Percutaneous transluminal coronary angioplasty
XPC: Xeroderma pigmentosum, complementation group C
